# Renal Effects of the Novel Selective Adenosine A_1_ Receptor Blocker SLV329 in Experimental Liver Cirrhosis in Rats

**DOI:** 10.1371/journal.pone.0017891

**Published:** 2011-03-10

**Authors:** Berthold Hocher, Susi Heiden, Karoline von Websky, Ayman M. Arafat, Jan Rahnenführer, Markus Alter, Philipp Kalk, Dieter Ziegler, Yvan Fischer, Thiemo Pfab

**Affiliations:** 1 Institute of Nutritional Science, University of Potsdam, Potsdam, Germany; 2 Center for Cardiovascular Research/Institute of Pharmacology, Charité, Berlin, Germany; 3 Department of Endocrinology, Diabetes and Nutrition, Charité Campus Benjamin Franklin, Berlin, Germany; 4 Department of Clinical Nutrition, German Institute of Human Nutrition, Potsdam-Rehbruecke, Nuthetal, Germany; 5 Department of Nephrology, Charité Campus Benjamin Franklin, Berlin, Germany; 6 Abbott Products GmbH, Hannover, Germany; Copenhagen University Hospital Gentofte, Denmark

## Abstract

Liver cirrhosis is often complicated by an impaired renal excretion of water and sodium. Diuretics tend to further deteriorate renal function. It is unknown whether chronic selective adenosine A_1_ receptor blockade, via inhibition of the hepatorenal reflex and the tubuloglomerular feedback, might exert diuretic and natriuretic effects without a reduction of the glomerular filtration rate. In healthy animals intravenous treatment with the novel A_1_ receptor antagonist SLV329 resulted in a strong dose-dependent diuretic (up to 3.4-fold) and natriuretic (up to 13.5-fold) effect without affecting creatinine clearance. Male Wistar rats with thioacetamide-induced liver cirrhosis received SLV329, vehicle or furosemide for 12 weeks. The creatinine clearance of cirrhotic animals decreased significantly (−36.5%, p<0.05), especially in those receiving furosemide (−41.9%, p<0.01). SLV329 was able to prevent this decline of creatinine clearance. Mortality was significantly lower in cirrhotic animals treated with SLV329 in comparison to animals treated with furosemide (17% vs. 54%, p<0.05). SLV329 did not relevantly influence the degree of liver fibrosis, kidney histology or expression of hepatic or renal adenosine receptors. In conclusion, chronic treatment with SLV329 prevented the decrease of creatinine clearance in a rat model of liver cirrhosis. Further studies will have to establish whether adenosine A_1_ receptor antagonists are clinically beneficial at different stages of liver cirrhosis.

## Introduction

Liver cirrhosis is often complicated by an impaired renal capacity of maintaining water and sodium balance. Splanchnic arterial vasodilatation due to an increased release of endogenous vasodilators leads to compensatory activation of the endogenous vasoconstrictor systems: the sympathetic nervous system, the renin-angiotensin system and the non-osmotic release of vasopressin [1]. This causes renal sodium/water retention and renal vasoconstriction eventually leading to the hepatorenal syndrome. In many patients, water retention can be controlled by sodium restriction and diuretics. However, diuretic therapy often entails deterioration of renal function [1]. There is an urgent clinical need for alternative pharmacological approaches.

The adenosine system with its four receptors (A_1_, A_2A_, A_2B_ and A_3_) is involved in several key functions of both liver and kidneys [2]. Decreasing the portal flow, as it is the case in cirrhosis, results in an activation of the hepatic adenosine system [3]. Adenosine, via A_1_ receptors (A_1_R), serves as a mediator for triggering the hepatorenal reflex leading to renal water and sodium retention [4], [5]. However, the exact localization of adenosine receptors within the liver remains unclear. In the kidney A_1_R are highly expressed in the preglomerular microcirculation, but also on the proximal tubules and other renal structures [6]. Short-term infusion of selective A_1_R antagonists inhibits the tubuloglomerular feedback and causes diuresis and natriuresis [7], [8]. Blockade of hepatic and renal A_1_R could therefore provide a new therapeutic option in conditions with sodium and water retention such as liver cirrhosis [9].

Thioacetamide has been widely used to induce chronic liver injury in animal models since it mimics the human disease closely. Rats treated with thioacetamide present with typical cirrhotic liver damage and an impaired ability of excreting sodium and water [10].

The first part of the present study evaluated the receptor binding affinity of the novel adenosine A_1_R antagonist SLV329 - a pyrimidine derivative - and confirmed its *in vivo* action on diuresis and natriuresis in healthy animals. As main hypothesis of this study, we tested whether SLV329 might exert diuretic effects without impairing renal function in animals with thioacetamide-induced liver cirrhosis.

## Results

### *In vitro* selectivity profile of SLV329

In receptor binding experiments using cloned human receptors SLV329 behaved as a potent (pK_i_ 9.2) and selective A_1_R ligand. The affinity of SLV329 for the other adenosine receptors was at least 100-fold lower (Table 1). The receptor-binding affinities and enzyme inhibitory properties of SLV329 were evaluated in a series of 94 receptors and 6 phosphodiesterases including adenosine transporters and a wide range of adrenergic, muscarinic, nicotinic, dopaminergic, serotoninergic, histaminergic, glutamatergic, opioid, angiotensin, bradykinin and neuropeptide receptors as well as ion channels and transporter sites. Only the significant affinities of SLV329 are summarized in Table 1. Apart from adenosine receptors, significant binding was measured only for the high-affinity rolipram-binding site on phosphodiesterase 4. This effect was consistent with the inhibition of this enzyme by SLV329. However, phosphodiesterase 4 inhibition was much less potent than A_1_R binding. The ratio of the K_i_ value for phosphodiesterase 4 inhibition by SLV329 to that of the A_1_R binding was 3170. The activities on other phosphodiesterases were at least 10-fold lower.

**Table 1:** Receptor binding affinities and enzyme inhibitory properties of SLV329.

Assay	Cell/tissue	Ligand	SLV329 Affinity
Adenosine A_1_	CHO cells	^3^H-DPCPX	9.2±0.2
Adenosine A_2A_	HEK293 cells	^3^H-CGS21680	7.2±0.2
Adenosine A_3_	HEK293 cells	^3^H-AB-MECA	6.9±0.1
Adenosine A_2B_	HEK293 cells	^3^H-DPCPX	6.3±0.1
Phosphodiesterase 4 rolipram binding	Total brain	^3^H-Rolipram	6.1±0.2
Phosphodiesterase 4 enzyme	U-937 cells	^3^H-cAMP	5.4±0.1
Phosphodiesterase 6 enzyme	Retina	^3^H-cAMP	4.3±0.1

Only significant affinities and enzyme inhibition of SLV329 are shown.

Cells and tissues used to obtain receptors/enzymes and radioactive ligands of the respective assays are listed. Cells and tissues were provided by Cerep (Celle l'Evescault, France)

Results are expressed as pK_i_ for radioligand affinity assays, and as pIC_50_ for enzyme inhibition. Mean ± standard deviation of at least 3 determinations.

The half-maximally inhibitory concentration (IC_50_) value of SLV329 is 3.2 µg/l.

### Effects of SLV329 on renal function in healthy rats

Five different intravenous doses of SLV329 were tested in healthy anesthetized rats. Table 2 shows that SLV329 treatment resulted in a marked dose-dependent increase of diuresis by up to 3.4-fold and sodium excretion by up to 13.5-fold compared to the time-matched vehicle control group (both p<0.0001). The half-maximally effective concentration (EC_50_) values for diuresis and natriuresis were calculated to be 26 µg/l and 17 µg/l respectively. In contrast, the rate of potassium excretion was only modestly affected (up to only 1.7-fold vs. vehicle). The creatinine clearance was not affected by SLV329, even at the highest dose. SLV329 caused a dose-dependent increase in urinary adenosine excretion by up to 4.2-fold.

**Table 2:** Effects of different doses of SLV329 in anesthetized rats.

SLV329	bolus (µg/kg)	Vehicle	18	54	180	540	945
	infusion (µg/kg*min)	Vehicle	0.37	1.1	3.7	11	19.3
Plasma SLV329 (µg/l)		0±0	11±2.6	31±4.4	54±12	86±21	182±63
Diuresis (fold vs. vehicle)		1±0.1	1.4±0.1*	2.5±0.3****	2.3±0.3***	3.4±0.4****	2.8±0.4****
Diuresis (ml/kg*h)		1±0.1	1.4±0.1*	2.5±0.3****	2.3±0.3***	3.4±0.4****	2.8±0.4****
Natriuresis (fold vs. vehicle)		1±0.2	5.0±0.8***	7.6±0.9****	7.9±1.3****	13.5±1.3****	8.3±1.4****
Natriuresis (µg/kg*h)		65±16	324±52***	497±62****	518±84****	882±87****	544±94****
Kaliuresis (fold vs. vehicle)		1±0.1	1.6±0.2	1.4±0.1	1.7±0.1**	1.3±0.1	1.6±0.2
Kaliuresis (µg/kg*h)		106±13	167±21	146±13	175±16**	136±12	170±22
Creatinine clearance (ml/min)		1.9±0.3	3.2±1.1	2.0±0.3	1.6±0.2	2.6±0.8	2.4±0.7
Adenosine excretion (nmol/h)		2.4±0.7	3.1±0.4*	4.9±0.8**	5.9±1.5*	10.2±2.3***	9.8±1.9***
Body weight (g)		363±3	360±2	356±3	362±2	364±2	357±2
Systolic blood pressure (mmHg)		107±6	108±2	104±3	110±5	113±7	116±6
Heart rate (beats/min)		313±19	317±23	328±21	350±10	294±23	301±30

A bolus of SLV329 followed by an intravenous infusion over 3 hours produces a strong dose-dependent stimulation of renal water, sodium and adenosine excretion without any effect on creatinine clearance. Diuresis and electrolyte excretion is expressed as fold stimulation vs. time-matched vehicle controls.

Data are means ± standard error of the mean.

n = 9-13 animals per dose.

*p<0.05,

**p<0.01,

***p<0.001,

****p<0.0001 vs. time-matched vehicle controls.

### Effects of SLV329 on renal function in thioacetamide-induced liver cirrhosis

**Figure 1:**
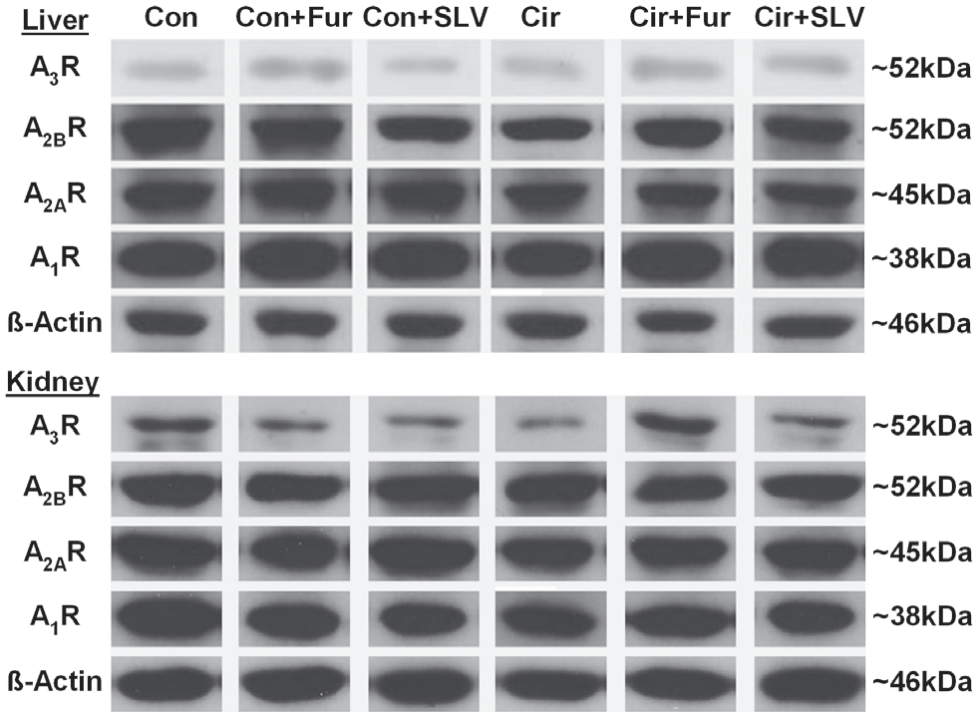
Representative immunoblots for hepatic and renal adenosine A_1_, A_2A_, A_2B_ and A_3_ receptors. Beta-actin was used for normalization before statistical analysis. Con, control; Cir, liver cirrhosis; Fur, furosemide; SLV, SLV329; kDa, kilodalton.

Descriptive data, results from histological evaluation and Western blots are summarized in Table 3. Representative immunoblots for hepatic and renal adenosine receptors are shown in Figure 1. The results of plasma and urine analyses are shown in Table 4.

**Table 3:** Effects of treatment with furosemide and SLV329 in rats with and without liver cirrhosis.

Group	Con	Con+Fur	Con+SLV	Cir	Cir+Fur	Cir+SLV
Bodyweight week 0 (g)	358±6	344±10	356±11	351±6	354±6	352±5
Bodyweight week 8 (g)	443±11	433±13	439±15	333±8***	345±6***	335±5***
Bodyweight week 16 (g)	464±11	448±10	479±16	299±9***	263±13***	296±9*** #
Water intake week 0–8 (ml/kg*d)	41±4	47±4	40±5	49±2*	46±1	50±2
Water intake week 8–16 (ml/kg*d)	65±2	69±2	70±4	44±2***	52±2*** §	50±2** §
Water intake week 11 (ml/kg*d)	63±4	76±5§	72±3§§	48±2**	52±2**	54±4** §
Systolic BP week 0 (mmHg)	124±2	126±6	130±3	126±1	125±2	127±2
Systolic BP week 12 (mmHg)	122±3	119±5	134±7§	110±5	118±5	118±3*
Liver weight (g)	12.5±0.5	11.9±0.5	13.2±0.7	13.9±0.5	13.4±0.5*	14.1±0.3
Kidney weight (g)	3.1±0.1	3.0±0.1	3.2±0.1	3.1±0.1	3.0±0.1	2.9±0.1
Liver interstitial fibrosis (%)	0.3±0.1	0.4±0.1	0.9±0.3	8.6±1.1***	5.2±1.1***	8.6±1.4***
Kidney interstitial fibrosis (%)	3.0±0.3	3.4±0.6	3.5±0.6	2.9±0.3	3.1±0.6	3.2±0.4
Kidney media/lumen ratio	3.3±0.4	3.1±0.2	2.6±0.3	2.6±0.2	2.8±0.2	3.0±0.3
Glomerulosclerosis index (0–4)	1.8±0.04	1.8±0.1	1.6±0.1	1.7±0.05	1.7±0.1	1.8±0.1*
Liver A_1_ receptor	1.0±0.1	1.0±0.2	1.1±0.1	0.8±0.1	1.0±0.1	1.0±0.1
Liver A_2A_ receptor	1.0±0.2	1.2±0.1	0.9±0.1	0.7±0.1	0.8±0.1	0.8±0.1
Liver A_2B_ receptor	1.0±0.2	1.0±0.3	0.7±0.1	0.4±0.1*	0.6±0.1	0.6±0.1
Liver A_3_ receptor	1.0±0.2	0.7±0.1	0.9±0.2	0.8±0.2	1.0±0.2	0.8±0.1
Kidney A_1_ receptor	1.0±0.03	1.0±0.2	0.6±0.1§§	0.8±0.1*	0.8±0.1	0.7±0.1
Kidney A_2A_ receptor	1.0±0.1	0.9±0.2	1.0±0.1	0.9±0.2	1.2±0.2	1.2±0.1
Kidney A_2B_ receptor	1.0±0.1	0.9±0.2	1.4±0.2	1.3±0.2	1.3±0.1	1.0±0.2
Kidney A_3_ receptor	1.0±0.1	0.7±0.2	0.8±0.2	0.7±0.1	1.2±0.2	0.9±0.3

Con, control; Cir, liver cirrhosis; Fur, furosemide; SLV, SLV329, BP, blood pressure.

Results of adenosine receptors from Western blots are expressed as fold expression vs. Con.

Data are means ± standard error of the mean. n = 8–14 per group.

*p<0.05,

**p<0.01,

***p>0.001 vs. Con group with same treatment,

§ p<0.05,

§§ p<0.01 vs. same group without Fur or SLV treatment,

# p<0.05 vs. same group with Fur treatment.

**Table 4:** Effects of treatment with furosemide and SLV329 on plasma and urine parameters in rats with and without liver cirrhosis.

Group	Con	Con+Fur	Con+SLV	Cir	Cir+Fur	Cir+SLV
ALT week 8 (U/l)	26±3	36±5	29±4	41±6	27±3* §	31±4
ALT week 16 (U/l)	55±4	48±2	47±4	73±9	66±11	53±7
Bilirubin week 8 (µmol/l)	1.3±0.1	1.5±0.3	1.4±0.2	7.3±0.7***	6.0±0.8***	5.7±0.9***
Bilirubin week 16 (µmol/l)	1.2±0.3	1.5±0.4	1.3±0.3	16±1.8***	22±2.9***	12±2.0*** #
Albumin week 8 (g/l)	31±0.4	32±0.4	31±0.2#	31±0.5	32±0.5	31±0.4
Albumin week 16 (g/l)	29±0.5	29±0.7	28±0.3#	26±0.6**	25±1.0*	26±0.5***
Creatinine week 8 (µmol/l)	52±2	54±3	54±3	55±2	52±2	49±1§
Creatinine week 16 (µmol/l)	60±1	60±2	55±1§§ ^#^	51±2*	47±2**	45±2** §
Urine volume week 8 (ml/d)	51±6	59±4	52±8	58±3	61±3	65±5
Urine volume week 16 (ml/d)	71±8	75±3	67±9	67±8	58±6	69±7
Na^+^ excretion week 8 (mmol/d)	3.2±0.5	3.1±0.2	2.9±0.5	3.6±0.5	3.7±0.4	2.9±0.3
Na^+^ excretion week 16 (mmol/d)	3.7±0.6	5.5±0.5	4.4±0.6	4.7±0.6	3.8±0.9	4.1±0.3
K^+^ excretion week 8 (mmol/d)	4.4±0.6	5.0±0.5	4.3±0.5	4.4±0.5	4.8±0.4	4.3±0.4
K^+^ excretion week 16 (mmol/d)	7.6±0.6	7.7±0.8	6.7±0.7	6.2±0.6	5.0±0.7	7.0±0.4

Con, control; Cir, liver cirrhosis; Fur, furosemide; SLV, SLV329.

In week 0, there were no significant differences between the groups, except for plasma creatinine (Con+Fur 46±6 vs. Cir+Fur 53±5 µmol/l; p<0.05) and urine volume (Con+Fur 70±16 vs. Cir+Fur 58±6 ml/d; p<0.05).

Data are means ± standard error of the mean. n = 8–14 per group.

*p<0.05,

**p<0.01,

***p>0.001 vs. Con group with same treatment,

§ p<0.05,

§§ p<0.01 vs. same group without Fur or SLV treatment,

# p<0.05 vs. same group with Fur treatment.

Livers of the thioacetamide-treated rats showed macroscopic signs of nodular cirrhosis and tended to be heavier than livers from control groups. While liver fibrosis was seen in thioacetamide-treated rats (p<0.001), kidney histology showed no relevant alterations in any of the groups. During the development of liver cirrhosis bilirubin rose markedly, whereas there was a significant decrease of plasma albumin. There was no relevant amount of ascites in any of the cirrhotic groups.

Bodyweight (p<0.001) and water intake (p<0.01) was lower in rats treated with thioacetamide as compared to control animals. Furosemide and SLV329 caused an increase of mean water intake (probably reflecting increased diuresis), particularly at the beginning of the treatment (e.g. week 11, p<0.05; Table 3).

**Figure 2:**
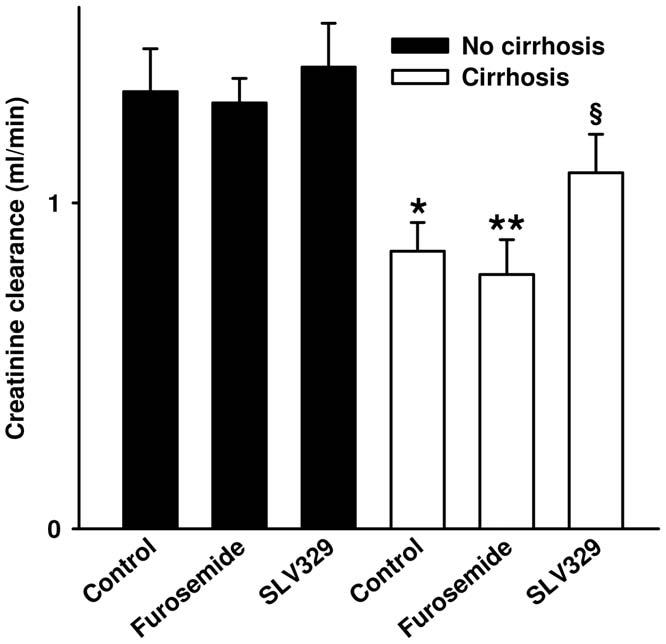
Creatinine clearance (week 16) in rats with and without liver cirrhosis. There were no significant differences between the groups in week 0 and week 8. In week 16 creatinine clearance of the cirrhotic SLV329 group was not significantly different from any of the non-cirrhotic groups. Data are means ± standard error of the mean. n = 8–10 per group (week 16). *p = 0.05, **p<0.01 vs. non-cirrhotic control rats. ^§^p = 0.07 vs. cirrhotic animals with furosemide treatment.

Plasma creatinine was reduced in all cirrhotic animals, most probably due to reduced muscle mass because of wasting. Plasma creatinine was significantly lower in the SLV329-treated groups, in spite of higher mean body weight. The creatinine clearance was significantly reduced in cirrhotic animals compared to controls (−36,5%, p<0.05; Figure 2). This was even slightly more pronounced in the group treated with furosemide (−41.9%, p<0.01 compared to controls). In contrast, the creatinine clearance of the cirrhotic group treated with SLV329 did not differ significantly from any of the non-cirrhotic groups.

**Figure 3:**
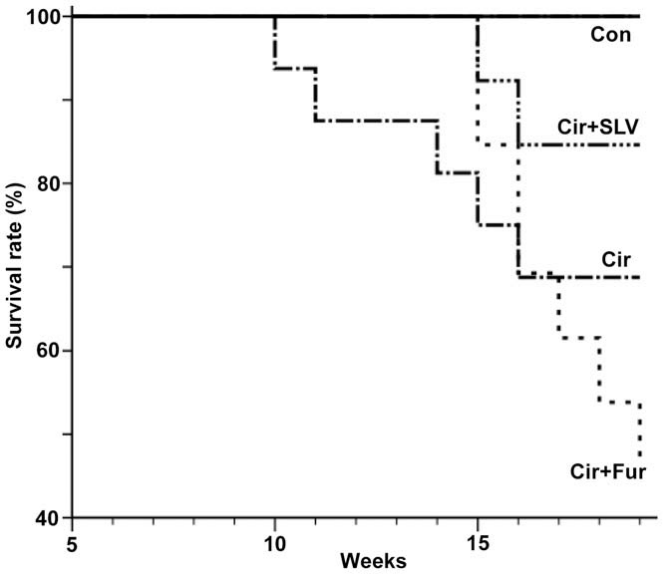
Kaplan-Meier mortality chart of rats with liver cirrhosis (Cir; mortality 5/14), cirrhotic rats with furosemide treatment (Cir+Fur; 7/13), cirrhotic rats with SLV329 treatment (Cir+SLV; 2/12) and all control groups without liver cirrhosis (Con; 0/24). p<0.05 for Cir+Fur vs. Cir+SLV (log-rank test).

The reduction of mortality in cirrhotic animals treated with SLV329 compared to untreated cirrhotic animals did not reach statistical significance. However, mortality was significantly lower compared to cirrhotic animals treated with furosemide (17% vs. 54%, p<0.05; Figure 3).

In the Western blot analyses hepatic A_2_R seemed to be reduced in cirrhotic rats. This difference was statistically significant for A_2A_R and A_2B_R when testing all cirrhotic rats vs. all controls (p<0.01). Treatment with SLV329 reduced A_1_R expression in renal tissue of non-cirrhotic animals (p<0.01).

Mean plasma concentrations of SLV329 were 49±8 µg/l on day 10 after the beginning of SLV329 treatment and 54±13 µg/l after 4 weeks in 6 random animals.

Heart rate, lipase, creatine kinase and urinary protein excretion were not different between the groups at any time (data not shown).

## Discussion

The present study was set out to investigate the receptor binding affinity of the novel A_1_R antagonist SLV329, to evaluate its in vivo effects on diuresis and natriuresis in healthy animals and to find out whether it exerts beneficial effects in an animal model of thioacetamide-induced liver cirrhosis.

Receptor binding experiments showed that SLV329 behaves as a potent and selective A_1_R ligand *in vitro*.

Intravenous treatment of healthy rats with SLV329 resulted in a strong dose-dependent diuretic and natriuretic effect, whereas the effect on kaliuresis was relatively small and the creatinine clearance remained unchanged. This is in line with effects that have been reported in a variety of rat strains including Wistar rats [11]–[13].

The ability of diuretics to prevent sodium reabsorption results in an increased delivery of electrolytes to the distal tubule. This leads, in turn, to an augmented release of adenosine, which may activate A_1_R in afferent arterioles [14]. By blocking those receptors, A_1_R antagonists cause an uncoupling of the tubuloglomerular feedback which may at least partly explain the elevated concentrations of adenosine measured in the urine of animals treated with SLV329 [15], [16]. However, urinary excretion of paracrine mediators do not necessarily reflect their local tissue concentrations. Alternatively, blockade of A_1_R might result in elevated intracellular cyclic adenosine monophosphate levels and release in the kidney, which will eventually lead to increased extracellular adenosine concentrations due to cyclic adenosine monophosphate degradation [17]–[19]. However, in spite of the observed increase in urinary adenosine excretion, SLV329 did not decrease the rate of creatinine clearance, even at the highest dose.

Previous studies demonstrated that a single application of an A_1_R antagonist causes an increase of renal sodium and water excretion in animals and patients with liver cirrhosis without affecting the glomerular filtration rate [20], [21]. As a next step towards a possible clinical application, the present study investigated for the first time the effects of a chronic application of a selective A_1_R antagonist on kidney function and mortality starting at an early stage of liver cirrhosis. This proof-of-concept experiment included a low-dose furosemide-treated group because loop diuretics are often applied in cirrhotic patients and tend to deteriorate renal function. However, monotherapy with a loop diuretic is of course not the typical clinical situation at an early stage of liver cirrhosis without severe water retention. In the present study the creatinine clearance, used as a surrogate for the glomerular filtration rate, was significantly reduced in cirrhotic animals, especially in those receiving furosemide. In contrast, the A_1_R antagonist SLV329 was able to prevent this decline of creatinine clearance. The reduction of mortality in cirrhotic animals treated with SLV329 in comparison to vehicle treatment was not statistically significant. However, mortality was significantly lower in cirrhotic animals treated with SLV329 in comparison to animals treated with furosemide (17% vs. 54%). It is a limitation of this study that creatinine clearance was used instead of inulin clearance. Inulin clearance was not used because mortality would have increased further due to additional anesthesia. Creatinine clearance is influenced by muscle mass, liver function and tubular secretion of creatinine. However, there were no significant differences of neither body weight nor liver function between cirrhotic animals with and without SLV329 treatment. Tubular secretion of creatinine increases with declining glomerular filtration rate. Thus, the glomerular filtration rate of untreated cirrhotic animals and those treated with furosemide will be even lower than the creatinine clearance suggests, making the difference to the group treated with SLV329 even larger.

Mean SLV329 plasma concentrations of 49–54 µg/l were actually reached by chronic application in the cirrhosis model. This plasma concentration was able to cause strong short-term effects on diuresis and saliuresis when administered intravenously as shown in Table 2. A steady-state is usually reached in the course of long-term diuretic therapy by means of compensatory mechanisms of tubular reabsorption [22]. This explains why the 24-hour urine volume and electrolyte excretion in week 16 is not different between the groups of the cirrhosis model. However, when looking at mean water intake over 8 weeks, as a putative surrogate of diuresis, the effects of furosemide and SLV329 can be detected. It would have been interesting to evaluate fractional sodium excretion and free water clearance. This was not done due to limited plasma quantities.

SLV329 does not affect the expression of hepatic or renal adenosine receptors in cirrhotic animals. The expression of A_2_R seems to be reduced in cirrhotic animals, independent of the treatment group. As yet, there are no reports on the expression of A_2_R in cirrhotic liver tissue. However, it is known, that hepatic A_2_R play an active role in the pathogenesis of hepatic fibrosis [23]. It is a limitation of this study that adenosine receptor expression was evaluated only in liver and kidney homogenates by Western blot. Immunohistochemistry or radioligand binding experiments might give further detailed information.

The beneficial effects of SLV329 in the cirrhosis model cannot be explained by morphological effects since SLV329 did not relevantly influence the degree of liver fibrosis, kidney histology or expression of hepatic or renal adenosine receptors. Animal studies suggest that liver cirrhosis activates the hepatorenal reflex via A_1_R, leading to renal water and sodium retention [24], [25]. Animal and human studies suggest that a resetting of the tubuloglomerular feedback contributes to the pathophysiology of kidney impairment in liver cirrhosis [26]. Thus, inhibition of both the hepatorenal reflex and the tubuloglomerular feedback might explain the higher rate of creatinine clearance in the animals treated with SLV329. In addition to the effect on creatinine clearance, yet unknown effects of SLV329 might contribute to the reduction of mortality. As an addition to the present study it would be interesting to study liver cirrhosis in the established murine A_1_R knockout model.

The ability of A_1_R antagonists to induce diuresis and natriuresis while not compromising glomerular filtration rate has become an attractive therapeutic option for the treatment of other fluid retention disorders, e.g. in kidney disease and heart failure, especially in conditions associated with diuretic resistance [27]. The preexisting experience with this class of drugs for other indications (including a large phase 3 trial) might facilitate the future translation of the results of this study to clinical application [28].

In conclusion, this study described some pharmacodynamic characteristics of the novel adenosine A_1_R-specific antagonist SLV329 and demonstrated its long-term safety and efficacy in an animal model of liver cirrhosis. Chronic SLV329 treatment starting at an early stage of liver cirrhosis prevented the decrease of creatinine clearance. Further studies will have to evaluate, whether SLV329 or other A_1_R antagonists are clinically beneficial at different stages of liver cirrhosis, either as an add-on to aldosterone antagonists or in combination with loop diuretics.

## Materials and Methods

### Receptor binding and enzyme assays

Receptor binding affinities as well as enzyme inhibitory properties of the new compound SLV329 were evaluated in a series of 94 receptors and 6 phosphodiesterases as described previously [29]. All cells and tissues needed for the assays were provided by Cerep (Celle l'Evescault, France).

Receptor binding assays were conducted as follows: after incubation of SLV329 with a receptor preparation and its radioactive ligand, the receptor preparations were rapidly filtered under vacuum through glass fiber filters, the filters were washed extensively with an ice-cold buffer using a harvester. Bound radioactivity was measured by scintillation counting using a liquid scintillation cocktail. Enzyme assays were carried out as follows: after incubation of SLV329 with an enzyme preparation and its radioactive substrate, radioactivity of the enzyme product was measured by scintillation counting using a liquid scintillation cocktail. Testing was done at a 3-log concentration range around a predetermined half-maximally inhibitory concentration (IC_50_) for the respective assay. The highest concentration tested for primes was 10 µM in receptor binding and 100 µM for enzyme assays. If no significant receptor binding or enzyme inhibition was detected at those concentrations SLV329 was considered to be inactive. Results were calculated as percentage of control values (enzyme assays) or for receptor binding assays as percentage of total ligand binding and that of nonspecific binding per concentration of SLV329. From the concentration-displacement curves IC_50_ values were determined by nonlinear regression analysis using Hill equation curve fitting. The inhibition constants (K_i_) were calculated from the Cheng-Prusoff equation K_i_ = IC_50_/(1+L/K_d_), where L is the concentration of radioligand in the assay and K_d_ the affinity of the radioligand for the receptor. Results were expressed as mean pKi values ± standard deviation (SD) of at least three separate experiments.

### Effects of SLV329 on renal function in healthy rats

All animal experiments of this study were conducted in strict accordance with the European Convention for the Protection of Vertebrate Animals used for Experimental and Other Scientific Purposes (ETS123) and the German law on animal welfare and all efforts were made to minimize suffering. The study protocol was approved by the Niedersächsisches Landesamt für Verbraucherschutz und Lebensmittelsicherheit (Bezirksregierung Hannover, approval number 509.6.42502-04/875).

Male Sprague-Dawley Crl:CD(SD)BR rats (150–170 g) were fasted overnight. The rats were anesthetized with 80 mg/kg thiobutabarbital intraperitoneally; additional doses of 40 mg/kg were given 2.5 h and 5 hours later. Catheters were placed in one jugular vein (for SLV329 or vehicle administration), one carotid artery (for blood sampling and blood pressure measurements), and the bladder. The rats were kept on a heated table to maintain body temperature at 37°C. After an equilibration period of 30 min, urine was sampled for a period of 3 h, then the animals received vehicle or SLV329 as follows: a slowly applied (30–60 s) loading bolus of 18, 54, 180, 540, and 945 µg/kg of SLV329 (in 0.45 ml/kg), followed by a continuous intravenous infusion at a rate of 0.37, 1.1, 3.7, 11, and 19.3 µg/kg SLV329 per minute (in 9.3 µl/kg per minute) for three hours. Urine and blood samples were collected 2 min before the SLV329 bolus and after three hours of SLV329 infusion. 5-sulfosalicylic acid was added to the urine aliquots for adenosine measurements at a final concentration of 8 g/l before freezing. Electrolytes and creatinine in plasma and urine were measured by standard automated analyzer by Medizinisches Labor Hannover (Hannover, Germany). Urine adenosine concentrations were quantified by high-pressure liquid chromatography using a MZ nucleosil C18 column (125×4 mm, 10 µm) with ultraviolet-detection (Immundiagnostik, Bensheim, Germany). Quantification of SLV329 plasma concentrations was performed after solid phase extraction using a validated reversed phase high-pressure liquid chromatography method with MS/MS-detection (Sciex Api 3000, Perkin Elmer, Waltham, MA, USA). The data describing the concentration-dependence of the diuretic and natriuretic effects of SLV329 were fitted to estimate the half-maximally effective concentration (EC_50_) using Prism 5.00 (GraphPad Software Inc., San Diego, CA, USA).

### Effects of SLV329 in thioacetamide-induced liver cirrhosis

The study protocol was approved by the Landesamt für Gesundheit und Soziales, Berlin (approval number G0163/06). Male Wistar rats (250–300 g) were maintained under controlled conditions (20±2°C, 12 h light/dark cycle) and kept on a standard diet (0.2% sodium) with water *ad libitum*.

Animals were divided into 6 groups:

1. Controls (Con; n = 8)
2. Controls with furosemide treatment (Con+Fur; n = 8)
3. Controls with SLV329 treatment (Con+SLV; n = 8)
4. Cirrhosis (Cir; n = 14)
5. Cirrhosis with furosemide treatment (Cir+Fur; n = 13)
6. Cirrhosis with SLV329 treatment (Cir+SLV; n = 12)

Liver cirrhosis was induced by oral administration of thioacetamide via drinking water for 18 weeks. The initial concentration was 0.03%. This concentration was modified weekly according to weight changes in response to thioacetamide. The concentration was increased/reduced by 0.015% (absolute) in case of weight gain/loss >25 g per week. Treatment with furosemide or SLV329 was started in week 8, since it is known that liver cirrhosis develops by then [30]. Thioacetamide concentrations remained unchanged from week 8 until week 12. In week 12 the concentration was increased by 0.015% in all animals receiving thioacetamide. The resulting concentration was given until week 18. Furosemide was injected intraperitoneally (7.5 mg/kg) thrice weekly (always between 8 and 10 a.m.) in the respective groups starting from week 8 until study end. Also starting from week 8 the respective groups received a standard rat chow formulated with SLV329. Chow was obtained from Altromin (Lage, Germany) in several concentrations (0.0075%, 0.014%, and 0.05% SLV329) and fed to the rats according to food intake. The target concentration was 5 mg/kg per day. Subsequently calculated mean intakes of the groups were Con+SLV 5.4 mg/kg*d and Cir+SLV 5.1 mg/kg*d. Blood was taken from the retro-orbital vein plexus 10 days and 4 weeks after the beginning of SLV329 treatment in 6 random animals to measure the plasma concentration of SLV329. The samples were analyzed as described above.

Body weight, water and food intake were measured weekly. Systolic blood pressure and heart rate were measured in week 0 and 12 by tail plethysmography as described previously.[31] All animals were placed in metabolism cages in week 0, 8 and 16. Blood was taken from the retro-orbital vein plexus. The animals were sacrificed after week 20 and liver and kidneys were excised. Kidney samples were embedded in paraffin, cut into 3 µm sections and submitted to periodic acid-Schiff, elastica and sirius red staining. The extent of glomerulosclerosis, the renal media/lumen ratio and renal interstitial fibrosis was determined as described previously [32]. The extent of liver fibrosis was analyzed as in the kidneys.

Alanine aminotransferase, bilirubin, albumin, creatinine, lipase and creatine kinase were measured in plasma using commercially available assays (ABX Pentra 400, Horiba Medical, Montpellier, France). Creatinine, sodium, potassium and protein were measured in urine also using ABX Pentra 400.

### Western blot

Western blot was performed in liver and kidney tissue for A_1_, A_2A_, A_2B_ and A_3_ receptors using a method based on a previous publication [33]. In brief, snap frozen liver and kidney samples were pulverized in liquid nitrogen and dissolved in lysis buffer. After incubation at room temperature for 10 min, the suspension was centrifuged (16.200 g, 20°C, 45 min). Protein concentrations were determined in the supernatant using the Bradford method. Supernatant and Bradford solution (40 µl each) were mixed in a well of a microtiter plate and incubated for 10 min at room temperature on a shaker. Extinction was then measured photometrically at 595 nm. The protein concentration of each sample was calculated according to a standard dilution series. The samples were diluted with lysis buffer (5 g/l) to assure equal loading. Samples (23 µg protein per lane) were separated by sodium dodecylsulfate polyacrylamide gel electrophoresis (10%, 80 V for 30 min, then 110 V) and semi-dry-blotted (1,5 mA/cm^2^, 60 min) onto nitrocellulose membranes. Ponceau staining of membranes confirmed equal loading of proteins. Membranes were blocked with 5% skim milk and incubated overnight with primary rabbit antibodies directed against A_1_R (0.025%, Sigma-Aldrich, Munich, Germany), A_2A_R (0.1%, Millipore, Schwalbach, Germany), A_2B_R (0.1%, Millipore), A_3_R (0.2%, Millipore), and beta-actin for normalization (0.0025%, Sigma-Aldrich). The specificity of the antibodies has been documented by the manufacturers and was not tested again in this study. After extensive washing blots were incubated with a horseradish peroxidase-linked anti-rabbit IgG (60 min, 0.0001%, Santa Cruz Biotechnology, Santa Cruz, CA, USA). Immunoreactive bands were detected using an enhanced chemiluminescence system and were subsequently quantified with the AlphaEaseFC software (Alpha Innotech, San Leandro, CA, USA). The values thus obtained were corrected for different conditions between single runs, using a standard dilution series that was run on each gel. Results of adenosine receptors were then normalized to beta-actin.

### Statistical analysis

Data was analyzed with SPSS 17.0 (SSPS Inc., Chicago, IL, USA). The nonparametric Kruskal-Wallis and the Mann-Whitney-U test were used to detect significant differences between groups of interest. Mortality rates were estimated by the Kaplan-Meier method and compared by log-rank test.
